# Laser Photocoagulation Subsequent to Intravitreal Ranibizumab for Retinopathy of Prematurity Long-Term Efficacy in Extremely Low Birth Weight Twins

**DOI:** 10.1155/joph/8213936

**Published:** 2025-09-10

**Authors:** Jin-Guo Chen, Xin Hong, Qing-Qing Huang, Ke-Xin Mo, Jing-Jin Zhang

**Affiliations:** ^1^Department of Ophthalmology, Fujian Maternity and Child Health Hospital, College of Clinical Medical College for Obstetrics & Gynecology and Pediatrics, Fujian Medical University, Fuzhou, Fujian, China; ^2^Department of Child Health Care, Fujian Maternity and Child Health Hospital, Fuzhou, Fujian, China; ^3^Department of Ophthalmology, The First Affiliated Hospital of Fujian Medical University, Fuzhou, Fujian 350005, China; ^4^Department of Ophthalmology, National Regional Medical Center, Binghai Campus of the First Affiliated Hospital, Fujian Medical University, Fuzhou, Fujian 350005, China

**Keywords:** extremely low birth weight, laser photocoagulation, ranibizumab, ROP, twins

## Abstract

**Purpose:** To evaluate the long-term efficacy of laser photocoagulation (LP) subsequent to intravitreal ranibizumab (IVR) for retinopathy of prematurity (ROP) in extremely low birth weight (ELBW) twins.

**Methods:** In this matched case–control retrospective study, ELBW (birth weight < 1000 g) twins developed Type I ROP or AROP who received IVR and LP consecutively (IVR + LP) grouped between July 2019 and May 2022 were reviewed. Primary structural outcome was assessed at 12 months postoperatively. Physical assessments, comprising ophthalmic, systemic, and neurodevelopmental evaluations were performed at a chronological age of 24–36 months. Children with spontaneous regression of ROP, matched by sex and age (±3 months) to the IVR + LP group in a 1:1 ratio, were defined as the control group. The independent *t*-test and Wilcoxon rank-sum test were used to analyze and describe the demography, morphology, and treatment outcomes.

**Results:** Twenty-four twins (48 eyes) with the mean gestational age (GA) and body weight (BW) at birth of 27.5 ± 1.7 weeks and 831.4 ± 140.9 g, respectively, were analyzed. Aggressive ROP in Zone I was observed in 8 eyes (16.7%). Stage 3+ disease was observed in 2 eyes (4.1%) in Zone I and 17 eyes (35.4%) in Zone II posterior. The median duration between IVR and LP was 197.5 days (range: 28–237 days). The reduction of cumulative clock hours (CCH) of ROP lesion showed a statistically significant difference after IVR (*p* < 0.05). The median laser spots count was 204 (range: 139–503), and the laser power ranged from 100 to 130 mW. Long-term follow-up physical examinations, including ophthalmic, systemic, and neurodevelopmental assessments, revealed no statistically significant differences between the IVR + LP and control groups. Notably, there were no differences in axial length (AL) and spherical equivalent (SE) (*p* < 0.05).

**Conclusion:** LP following IVR treatment provides long-term effective outcomes for ROP in ELBW twins, without affecting ophthalmic, systemic, or neurological development, and reduces the risk of myopia compared to LP monotherapy.

## 1. Introduction

Retinopathy of prematurity (ROP) is a retinal vascular proliferative disorder affecting premature and low birth weight infants, making it a leading cause of childhood blindness globally [1]. The current primary treatments for ROP include laser photocoagulation (LP) and intravitreal injections of antivascular endothelial growth factor (VEGF), such as ranibizumab [2–4]. LP works by ablating the peripheral avascular retina, thereby improving the hypoxic state of the retina by reducing oxygen demand in the ROP ridge. This process results in decreased VEGF levels and promotes revascularization [5]. Intravitreal ranibizumab (IVR), on the other hand, decrease VEGF concentration and inhibit the growth of new blood vessels. While both treatments are effective, each has significant limitations when used alone. LP, though reducing the risk of retinal detachment [6], can cause peripheral vision loss, narrower visual field, and high myopia [7, 8]. Although IVR targets the angiogenic process more directly, VEGF, as an independent class of angiogenesis-related factors, is also widely expressed in vital organs, such as the heart and brain. Chen et al. reported that serum VEGF levels were suppressed for at least 7 days following IVR [9]. Several studies have investigated the potential adverse effects of anti-VEGF injections in human neonates, specifically neurodevelopmental delays [3, 10]. Therefore, it is essential to consider the potential systemic effects and neurodevelopmental risks associated with VEGF inhibition. Additionally, IVR alone may cause ROP reactivation, requiring multiple injections and increasing the risks of complications such as retinal detachment and endophthalmitis. Preliminary studies suggest that combining LP and IVR may provide a synergistic effect [11], effectively inhibiting pathological angiogenesis and potentially reducing the recurrence of ROP, thus enhancing the long-term efficacy of treatment [12]. Furthermore, the combined treatment may mitigate the drawbacks associated with each modality, such as reducing the number of required IVR injections and preserving more peripheral vision compared to LP alone.

The incidence of ROP is particularly high in extremely low birth weight (ELBW) infants (< 1000 g at birth) and is even more pronounced in ELBW twins, largely due to advancements in reproductive technology and neonatal resuscitation techniques [13, 14]. Due to the lower body weight (BW) and gestational age (GA) at birth, twins are more likely to experience systemic complications and require longer periods of oxygen therapy compared to singletons [15]. The specialized and meticulous care required for these infants underscores once again that treating ELBW twins with ROP presented a significant challenge. However, there are few reports on the treatment of this unique population. The long-term efficacy of combined LP and IVR in ELBW twins has not been extensively studied. Given the high risk and unique characteristics of this population, it is crucial to investigate this treatment modality to improve outcomes and prevent long-term visual impairment.

This study aimed to evaluate the structural integrity of the retina following LP subsequent to IVR in the treatment of ROP in ELBW twins, and to assess the long-term functional outcomes, including ophthalmic, systemic, and neurodevelopmental assessments, at least 2 years post-treatment.

## 2. Methods

This retrospective review study was carried out in ophthalmologic departments affiliated with Fujian Maternity and Child Health Hospital from July 2019 to May 2022. Written informed consent was obtained from the guardians of all patients. This study adheres to the Declaration of Helsinki and received IRB approval (IRB No. 2022KYLLRD01046, 2024KY072). Infants with ELBW (< 1000 g), who were twins and diagnosed with type 1 ROP or AROP [16], were included in this study. Infants with other retinal diseases, such as Coats' disease and familial exudative vitreoretinopathy, or those with incomplete records or lost to follow-up, were excluded.

The first screening was conducted either 4 weeks postnatal age or at a postmenstrual age (PMA) of 31 weeks, whichever occurred first. Details of the infants, including gender, BW and GA at birth, PMA at screening, zone and stage of ROP, presence of aggressive retinopathy of prematurity (AROP), plus disease (characterized by tortuosity and dilation of retinal vessels), hemorrhage on the fibrovascular ridge, and cumulative clock hours (CCH) of ROP lesion, were documented. Each preterm infant was diagnosed using fundus imaging with the RetCam III Imaging System (Clarity Medical Systems Inc., Pleasanton, CA). The diagnosis was rechecked and confirmed using binocular indirect ophthalmoscopy with scleral depression, as needed.

All eligible infants received IVR, with injections administered 1.5 mm posterior to the limbus. A 0.25 mg/0.025 mL dose of ranibizumab (Novartis, Basel, Switzerland) was injected using a sterile 30 G 0.5 inch needle. New equipment was used for the other eye to minimize the risk of infection. Postoperative follow-ups were conducted at 2 days, 1 week, and 2 weeks, with additional follow-up visits scheduled based on the progression of retinal vasculogenesis. At each visit, all infants underwent dilated indirect ophthalmoscopy, and wide-field imaging was captured using the RetCam III Imaging System. LP was performed for cases of reactivation or peripheral avascular areas (PAR) greater 2 prism diopters (PD) more than 6 months after IVR. An 810-nm diode laser (SUPRA 810, France) was used, delivered through a 28D binocular indirect ophthalmoscope under general anesthesia, to perform photocoagulation. The photocoagulation area covers the avascular zone between the ora serrata and the ridge, excluding the ridge itself. The laser spot energy was set between 100 and 130 mW, with an exposure time of 200 ms, and coagulation points spaced at 0.5 spot diameter intervals. Grade III spot intensity was employed to achieve a grey-white retinal reaction during the procedure.

A favorable outcome was defined as regression of the ridge and plus disease, with continued vascular extension into the peripheral retina [17]. A negative outcome was defined as worsening ROP after treatment, such as tractional retinal detachment or temporal dragging of the fovea. All fundus screenings, IVR injections, and laser treatments were performed by the same experienced surgeon.

**Figure 1:**
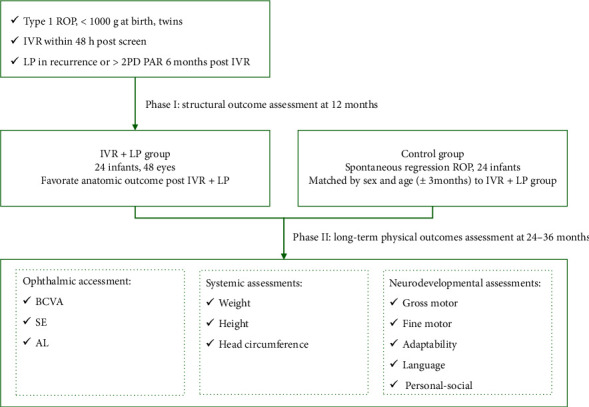
Study flowchart.

Physical examinations, including ophthalmic, systemic, and neurodevelopmental assessments, were performed on all children at 24–36 months postoperatively (Figure 1). Considering that age and sex significantly influence infants' ocular and overall development conditions, premature infants with spontaneously regressed ROP (control group) were matched by sex and age (±3 months) to the IVR + LP group in a 1:1 ratio. Data from the right eyes of subjects were selected for statistical analysis. Ophthalmic assessments included best corrected visual acuity (BCVA), axial length (AL), and spherical equivalent (SE) after cycloplegic refraction. BCVA was assessed using the HOTV test chart (Cat. No. 2014) for testable children and was converted to logMAR for statistical analysis [18]. Uncooperative children were tested for their ability to fix and follow a 5-cm toy [19]. AL values were obtained using the optical biometer AL-Scan (Nidek Co., Ltd., Japan) [20]. Measurements were performed with pupils in a nondilated state, and each eye underwent three successive measurements during the procedure. Resistant children could be engaged with playful strategies, such as pretending the device contained cartoons. After the examination, the AL signal curve was checked, with excessive noise indicating the need for a repeat measurement. Cycloplegic refraction was measured using a retinoscopy (YZ24; 66 Vision-Tech, China). Cycloplegia was induced using 1% cyclopentolate hydrochloride eye drops (Alcon, Switzerland), applied to both eyes three to four times at 10-min intervals. Completion of cycloplegia was confirmed when the pupillary light reflex disappeared and the pupil diameter exceeded 6 mm. SE was calculated as the spherical refraction plus half of the cylindrical refraction.

Systemic evaluation included measurements of BW, height, and head circumference. Neurodevelopmental testing at 24–36 months was conducted using the Gesell Developmental Diagnosis Scale (GDDS), T which assesses children aged 0–3 years across five domains: gross motor, fine motor, adaptability, language, and personal-social. The developmental quotient (DQ) was defined as 100 × (developmental age/actual age) [21].

### 2.1. Statistical Analysis

Data analysis was conducted using SPSS version 26.0 (IBM Corp., Armonk, NY, USA). Normally distributed measurement data were represented as the mean ± standard deviation (SD) and compared using the independent *t*-test. Non-normally distributed measurement data were represented as the median (P50) with interquartile range (P25 to P75) and analyzed using the Wilcoxon rank-sum test or paired samples Wilcoxon signed rank test. A *p* value < 0.05 was considered statistically significant.

## 3. Results

**Figure 2:**
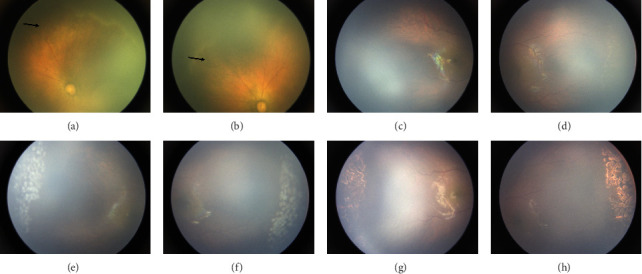
Infant Number 11 (BW: 735 g, GA: 26 weeks at birth) presented with AROP located in Zone I in both eyes. (a, b) Abnormal vascular anastomoses were observed in the posterior pole before treatment (black arrow). (c, d) 6 months post-IVR, PAR > 2PD and abnormal vascular branching was observed. (e, f) Grade III laser spots were evenly distributed in the PAR, with each spot spaced 0.5 spot diameters apart. (g, h) The laser spots formed well and fused in long-term follow-up (4 months) post-LP.

**Figure 3:**
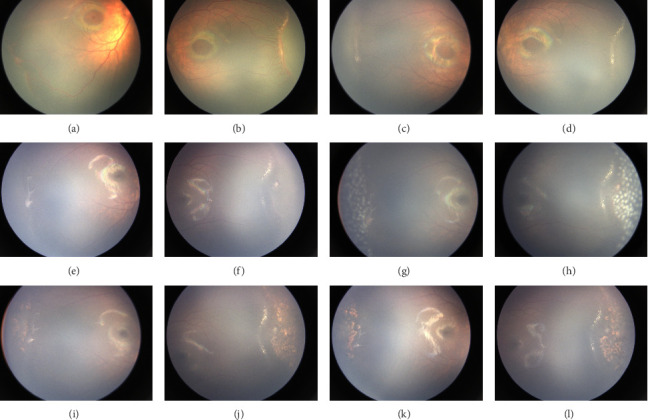
Infant Number 15 (BW: 968 g, GA: 28 weeks at birth) presented with Stage 3 located in Zone II in both eyes. (a, b) Pre-IVR, both eyes exhibited abnormal vascular anastomoses and hemorrhage on the fibrovascular ridge with fibrous proliferation changes. (c, d) The proliferative membrane showed regression, and the vessels crossed over the original ridge and continued growing. (e, f) A proliferative membrane was identified on the temporal side of the OD, and ridge changes were observed in the OS. (g, h) LP was applied to the PAR on the temporal sides of both eyes. (i–l) Regression of the fibrous proliferative membrane in OD and the ridge in OS after LP.

This study included 24 infants (48 eyes) who met the inclusion criteria (Table 1), of whom 12 (50%) were male. The mean BW at birth was 831.4 ± 140.9 g (range: 480–900 g), and GA at birth was 27.5 ± 1.7 weeks (range: 23–29 weeks). At presentation, AROP in Zone I was observed in 8 eyes (16.7%) (Figures 2(a) and 2(b)). Stage 3+ disease was observed in 2 eyes (4.1%) in Zone I and 17 eyes (35.4%) in Zone II posterior. The remaining 21 eyes (43.8%) were classified as Zone II stage 2+. The median CCH of ROP was 8 (range: 5–12) before treatment. All the eyes exhibited plus disease, and a total of 22 eyes (45.8%) presented with hemorrhage on the fibrovascular ridge (Figures 3(a) and 3(b)), an additional risk factor for poor outcomes.

**Figure 4:**
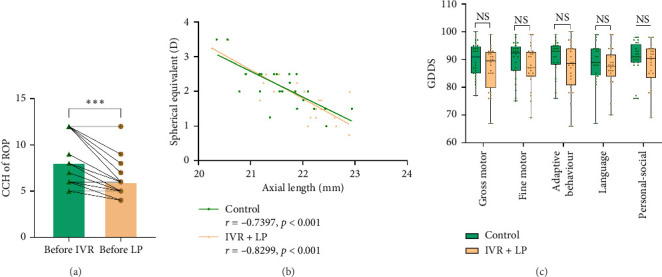
(a) The reduction of ROP CCH after IVR showed a statistically significant difference. ^∗∗∗^*p* < 0.001. (b) A notable negative correlation between SE and AL in the two group. (c) Gross motor, fine motor, adaptive behavior, language, and personal-social assessed by GDDS. Ns: *p* > 0.05.

Infants accepted IVR within 48 h have been diagnosed with type 1 ROP or AROP. During the follow-up period, LP was performed for cases of reactivation or PAR greater than 2 PD more than 6 months after IVR. Combined treatment was administered in all infants in this study. Six infants (12 eyes) required LP for reactivation, and 18 infants (36 eyes) underwent LP for PAR. The median interval between IVR and LP was 197.5 days (range: 28–237 days). The reduction of CCH of ROP showed a statistically significant difference after IVR (Figure 4(a)). The median laser spot count was 214 (139–503), and the laser power used in this study was in the range from 100 to 130 mW (Table 1).

Infant number 11, with a BW of 735 g and GA of 26 weeks at birth (Table 1, Figure 2), presented with AROP in Zone I in both the right eye (OD) and left eye (OS). Abnormal vascular anastomoses were observed in the posterior pole. IVR was implemented in both eyes simultaneously (PMA: 39 weeks). Peripheral vascular growth was observed in both eyes 6 months post-IVR compared to the preoperative status. However, a large avascular area (> 2PD) remained in the periphery, and abnormal vascular branching were presented. Consequently, uniform LP was applied to the PAR on the temporal sides of both eyes (OD: 238 and OS: 278 spots). The laser was carefully focused on the retina, and laser spots were evenly distributed in the PAR, with each spot spaced 0.5 spot diameters apart. A gray-white retinal reaction, defined as a grade III spot, indicated a satisfactory spot. At 4 months post-LP, the laser spots in both eyes were observed to have formed well and fused effectively.

Infant number 15, with a BW of 968 g and GA of 28 weeks (Table 1, Figure 3), presented with Stage 3 in Zone II in OD and OS. Pre-IVR, both eyes exhibited abnormal vascular anastomoses and ridges with fibrous proliferation changes. IVR was performed (PMA: 38 weeks). The proliferative membrane in both eyes showed regression, and the vessels crossed over the original ridge and continued growing 2 weeks post-IVR, indicating a potentially favorable outcome. Regrettably, a proliferative membrane reappeared on the temporal side of OD approximately 2 months post-IVR, which could potentially lead to tractional retinal detachment. Additionally, ridge changes reappeared in OS, indicating signs of reactivation. Consequently, both eyes underwent LP, which was applied to the PAR on the temporal sides of both eyes (OD: 175 and OS: 164 spots). Regression of the fibrous proliferative membrane in OD and the ridge in OS were observed 2 weeks post-LP, becoming more pronounced 6 months post-LP.

Notably, all children achieved favorable anatomical outcomes: plus disease regressed, and PAR < 2PD. The size of the laser scars gradually increased over time but did not extend into Zone I during long-term follow-up (Figures 2(e), 2(f), 3(k), 3(l)). Preretinal hemorrhage was observed in some cases but resolved spontaneously without intervention. No long-term sequelae, such as cataracts, vitreous hemorrhage, late retinal detachments, glaucoma, or macular anomalies, were observed. Additionally, no systemic adverse reactions were reported.

The mean chronological age at the time of physical examination was 29.8 ± 2.5 months (range: 26–36 months) in the control group and 28.8 ± 6.9 months (range: 24–36 months) in the LP + IVR group, with no significant difference between the two groups. As shown in Table 2, a significant difference in BW and GA at birth was observed between the two groups. However, no significant differences were found in physical development parameters, including weight, height, or head circumference, between the two groups (*p* > 0.05) during at least 2 years of follow-up.

Additionally, there was no statistically significant difference in ophthalmic outcomes between the two groups at a chronological age of 24–36 months. Due to the limited cooperation of pediatric patients during examinations, the control group consisted of 17 patients, while the IVR group consisted of 16 patients who underwent the HOTV test chart examination. The remaining children passed the fixation test (fix and follow a 5-cm toy). The mean BCVA (logMAR) in the control group was 0.3 ± 0.7, while the average BCVA in the IVR + LP group was 0.4 ± 0.1 (*p*=0.172). The mean SE was +2.1 ± 0.6 D (range: +1.00 to +3.50 D) in the control group and +1.9 ± 0.7 D (range: +0.75 to +3.50 D) in the IVR + LP group. The mean AL was 21.7 ± 0.6 mm (range: 20.35–22.94 mm) in the control group and 21.9 ± 0.7 mm (range: 20.26–22.90 mm) in the IVR + LP group. In the IVR + LP group, the SE (*p*=0.086) indicated a more myopic refractive error, and the AL (*p*=0.140) was longer compared to the control group (Table 2). However, these differences were not statistically significant (Table 2). A notable negative correlation between SE and AL was observed in both groups (Figure 4(b)).

During the same time period, neurodevelopmental testing using the GDDS was conducted for both groups of pediatric patients. The assessment included five components: gross motor, fine motor, adaptability, language, and personal-social. As shown in Figure 4(c) and Table 2, no significant differences were observed between the control group and the IVR + LP group (all *p* > 0.05).

Notably, Cases 19 and 20 were extremely premature twins (GA: 23.7 weeks; BW: 480 and 500 g) who presented with AROP in Zone I in both eyes. Both cases showed ROP reactivation 28 days after initial IVR and subsequently required LP treatment. At the 2.5-year follow-up examination, these twins demonstrated similar visual outcomes with BCVA of 0.5 logMAR in both cases, accompanied by shorter ALs (20.58 mm for Case 19 and 20.26 mm for Case 20) and hyperopic refractive errors (+3.25 D and +3.50 D, respectively). Their physical growth parameters, including BW (14.5 and 14.3 kg), body height (96 and 99 cm), and head circumference (48 and 47.5 cm), as well as the GDDS score (gross motor: 92 and 90; fine motor: 86 and 87; adaptive behavior: 95 and 94; language: 92 and 94; personal-social: 95 and 93), all fell within normal ranges.

## 4. Discussion

To the best of our knowledge, this was the first study on the long-term treatment outcomes of ROP in ELBW twins. Previous studies have reported that ELBW twins are at a heightened risk of developing ROP [14]. This risk is compounded by the challenges associated with the prenatal and postnatal growth and development of twins. ELBW twins experience shared intrauterine conditions, such as intrauterine growth restriction [22], and shared placental circulation, which impacts nutrient and oxygen delivery [23]. As shown in the current study, all patients (100%) exhibited plus disease and 22 eyes (45.8%) presented with hemorrhage on the fibrovascular ridge. Additionally, AROP in Zone I was observed in 8 eyes (16.7%). Stage 3+ disease was observed in 2 eyes (4.1%) in Zone I and 17 eyes (35.4%) in Zone II posterior.

LP or IVR monotherapy may lead to the recurrence or rebound of ROP despite initial successful treatment [24–26]. Dwivedi et al. reported that the outcome of laser treatment was significantly influenced by the disease zone [27], with Zone I posterior disease exhibiting the worst outcomes, resulting in 78.6%–100% unfavorable outcomes [28]. Moreover, several studies have demonstrated peripheral retinal avascularization following intravitreal anti-VEGF administration in eyes with ROP [29–31].

Previous studies have demonstrated that the combination of intravitreal injection of anti-VEGF along with LP promoted excellent outcomes [28, 32]. Parchand et al. reported that in their series involving combined IVR and LP for Zone I ROP, all eyes exhibited prompt regression of neovascularization, with no recurrence observed [33]. The potential benefits of combined therapy may arise from the synergistic effects of anti-VEGF injections and laser treatment, as observed in proliferative diabetic retinopathy. This approach could result in less intense laser applications and a reduction in the number of intravitreal injections and follow-up visits. In our study, the median CCH of ROP associated with ROP decreased from 8 prior to IVR administration to 5 at the time of LP (Figure 4(a)), suggesting that the reduction in areas requiring laser treatments was due to vascular expansion following IVR. Fortunately, this study reported no instances of retinal detachment or need for vitrectomy intervention, and there were no cases of blindness. These favorable outcomes may also be attributed to early screening of infants based on Chinese criteria, which allows most ROP diagnoses to be made before the onset of fibrovascular proliferation. Proactive early intervention is also crucial. We recommend initiating active treatment at stage 2+, as delaying treatment until stage 3+ may reduce efficacy and necessitate treatment of a larger area with LP. Additionally, the median duration between IVR and LP was 197.5 days (range: 28–237 days), which was longer than previously reported [17]. In this study, PAR was followed for up to 6 months post-IVR unless reactivation occurred. This extended duration, which allowed retinal vessels more time to grow, may have contributed to the significantly lower number of laser spots and a reduction in laser-related complications, such as myopia.

Long-term treatment outcomes of combining IVR and LP were carefully monitored. Assessments included ophthalmologic examinations, systemic indices, and neurological evaluations, conducted at a chronological age of 24–36 months. Among cooperative patients, no significant differences in BCVA were observed between the two groups, suggesting that IVR + LP treatment offers a favorable visual prognosis [34], consistent with previous reports [6, 19]. In the current study, a notable negative correlation between SE and AL was observed in both groups, with the degree of hyperopia decreasing and the degree of myopia increasing over time (Figure 4(b)). These findings align with both domestic and international research on the relationship between refractive development and ocular biometric parameters in normal children. Numerous studies have demonstrated that in patients undergoing laser treatment, AL does not increase significantly [6, 35–37]. The differences in AL between the two groups were not statistically significant, which is consistent with findings in previous reports. The mean SE in the IVR + LP group was +1.9 ± 0.7 D (range: +0.75 to +3.50 D), compared to +2.1 ± 0.6 D (range: +1.00 to +3.50 D) in the control group. Statistical analysis revealed no significant differences in SE between the two groups. These findings contrast with previous studies that suggested IVR combined with LP may carry a higher risk of myopia. In Lu's study, the mean SE was differed significantly between the groups, with −2.43 ± 3.56 D in the laser group and −0.53 ± 3.12 D in the injection group [6]. Similarly, Ruan et al. reported that, compared to spontaneous regression of ROP, children who received laser treatment often experienced more severe refractive errors, including astigmatism, high myopia, and anisometropia [38]. The observed differences in SE in the present study may be attributed to infants receiving LP treatment subsequent to IVR. The CCH of ROP was reduced to a specific value between the time of IVR and LP (Figure 4(a)). Consequently, the laser spot count ranged from 139 to 503, which was lower than previously reported values [34], suggesting that initial IVR treatment can reduce the extent of the ROP lesion and optimize subsequent LP treatment. This observation aligned with the insights from Alqurashi and Tafadzwa, who proposed that the stage and zone of ROP exert a more significant influence on myopia development than LP itself. Furthermore, the severity of myopia following LP appeared to increase with a higher number of laser burns or more extensive retinal treatment [39, 40]. The laser power used in our study ranged from 100 to 130 mW, significantly lower than values typically reported in previous studies [8]. Lower laser power was considered another crucial factor in reducing the risk of myopia, a perspective validated by Hoppe et al. [41], who used similar laser power levels and reached the same conclusion. Therefore, we recommend using low-energy settings to achieve effective Grade III spots during retinal laser procedures, as this approach maximizes therapeutic efficacy while minimizing potential complications. Achieving effective Grade III laser spots requires precise focusing of the laser on the retina. In this study, the relatively mild severity of myopia following LP after IVR was likely due to the limited extent of the laser-treated area. Consistent with prior reports, laser treatment in Zone I was associated with an increased risk of myopia, whereas laser treatment in Zone II did not carry the same risk [42].

Increasing attention has been directed toward the potential systemic effects of anti-VEGF drugs injected into the vitreous cavity, as they can reduce systemic VEGF levels [43], potentially impacting the central nervous system and other organ systems [10]. Murakami et al. found comparable neurodevelopmental outcomes between intravitreal bevacizumab (IVB) and LP treatments for ROP, showing no statistically significant differences in childhood developmental delay rates or IQ scores at 5-year follow-up [44]. Regarding the pharmacodynamic profiles of anti-VEGF agents, both IVR and IVB demonstrate temporary suppression of systemic VEGF levels in ROP patients. However, important pharmacokinetic differences exist—ranibizumab's relatively brief plasma half-life results in substantially reduced systemic VEGF inhibition following ocular administration compared to bevacizumab [30]. This pharmacokinetic advantage may explain the favorable long-term visual and developmental outcomes observed in our most extreme cases (Cases 19 and 20, birth weight 480 and 500 g, respectively). In the current study, although significant differences in BW and GA at birth were observed (*p*=0.001), no significant differences were found in BW (*p*=0.608), height (*p*=0.737), or head circumference (*p*=0.279) between the IVR + LP group and the control group at a chronological age of 24–36 months. This outcome was consistent with the findings reported by Stahl et al. [19]. The GDDS was selected to assess neurodevelopment. Systemic complications were more common in ELBW twins, warranting heightened attention and careful monitoring. Encouragingly, the children showed no abnormalities in gross motor, fine motor, adaptability, language, and personal-social compared to the control group (Figure 4(c)). The results confirmed that a 0.25 mg/0.025 mL dose of ranibizumab was safe and effective for ELBW twins with ROP.

The primary limitation of this study was its small sample size, which was attributable to the low prevalence of ELBW twins (birth weight < 1000 g) who developed type I ROP or AROP. Nonetheless, the study provided valuable preliminary insights. Although long-term follow-up indicated satisfactory outcomes, prolonged monitoring is required to evaluate potential late-onset ocular or systemic effects of IVR combined with LP. Future multicenter studies with larger cohorts and extended follow-up periods are necessary to validate and expand these findings.

## 5. Conclusion

LP following IVR proved to be a beneficial treatment protocol for ELBW twins with Type 1 ROP or AROP. This approach potentially reduced the risk of myopia, while providing excellent long-term outcomes without adversely affecting systemic or neurodevelopmental evaluations.

## Figures and Tables

**Table 1:** Characteristics of extremely low birth weight twins with ROP.

N	Sex	BW at birth (g)	GA at birth (weeks)	PMA at primary IVR (weeks)	Eye	Zone	Stage	Plus disease	Hemorrhage on fibrovascular ridge	ROP CCH	Interval between IVR and LP (days)	ROP CCH when LP	Laser spot count	Laser power	Outcome
1	M	961	26.3	36.3	OD	II	2	+	−	5	189	4	178	110	1
					OS	II	2	+	−	5	189	4	160	110	1

2	M	892	28.2	34.6	OD	II	2	+	−	6	195	4	150	110	1
					OS	II	2	+	−	6	195	4	176	110	1

3	M	993	28.2	36.3	OD	II	2	+	+	5	202	5	150	110	1
					OS	II	2	+	+	5	202	5	139	110	1

4	M	923	29	42.5	OD	II	2	+	+	6	48	5	180	120	1
					OS	II	2	+	+	6	48	5	190	120	1

5	F	974	29	35.5	OD	II	3	+	−	7	216	5	203	110	1
					OS	II	3	+	−	7	216	5	234	110	1

6	F	873	29	36.5	OD	II	2	+	−	6	207	6	220	120	1
					OS	II	2	+	−	6	207	6	240	120	1

7	F	736	26.3	38.1	OD	I	3	+	+	12	45	8	267	120	1
					OS	I	3	+	+	12	45	8	249	120	1

8	F	785	26.3	36.1	OD	II	2	+	−	12	209	6	198	110	1
					OS	II	2	+	−	12	209	6	185	110	1

9	F	975	29.1	36.4	OD	II	3	+	−	6	219	4	179	110	1
					OS	II	3	+	−	6	219	4	148	110	1

10	F	785	29.1	36.4	OD	II	3	+	−	7	228	5	168	110	1
					OS	II	3	+	−	8	228	5	197	120	1

11	F	735	26.1	39.1	OD	I	AROP	+	+	12	180	9	205	120	1
					OS	I	AROP	+	+	12	180	9	216	120	1

12	F	721	26.1	39.1	OD	I	AROP	+	−	12	180	7	190	120	1
					OS	I	AROP	+	−	12	180	7	220	120	1

13	M	814	26.2	39	OD	II	3	+	+	8	180	6	179	120	1
					OS	II	3	+	+	8	180	6	205	130	1

14	M	787	26.2	39	OD	II	3	+	−	7	197	5	169	120	1
					OS	II	2	+	−	6	197	5	208	130	1

15	M	963	28	38	OD	II	3	+	+	9	198	6	175	120	1
					OS	II	3	+	+	9	198	6	164	120	1

16	M	954	28	38	OD	II	3	+	−	7	198	5	156	110	1
					OS	II	3	+	−	7	198	5	149	110	1

17	M	734	29.4	41.5	OD	II	3	+	+	7	66	5	207	110	1
					OS	II	2	+	+	7	66	5	245	120	1

18	M	735	29.4	42.2	OD	II	3	+	+	9	54	6	237	120	1
					OS	II	2	+	+	9	54	6	256	120	1

19	M	480	23.7	34.8	OD	I	AROP	+	+	12	28	12	487	120	1
					OS	I	AROP	+	+	12	28	12	496	130	1

20	M	500	23.7	34.8	OD	I	AROP	+	+	12	28	12	486	120	1
					OS	I	AROP	+	+	12	28	12	503	130	1

21	F	900	27.4	35.1	OD	II	2	+	−	8	200	6	245	120	1
					OS	II	2	+	−	8	200	6	231	120	1

22	F	860	27.4	35.1	OD	II	2	+	−	5	226	4	177	110	1
					OS	II	2	+	−	5	226	4	165	110	1

23	M	970	29.4	35.7	OD	II	2	+	−	7	220	5	205	120	1
					OS	II	2	+	−	7	220	5	223	110	1

24	M	950	29.4	33.7	OD	II	3	+	+	8	237	5	246	130	1
					OS	II	3	+	+	8	237	5	261	110	1

Mean (SD) or M (P25, P75) Range		831.4 (140.9) 480 to 990	27.5 (1.7) 23.7 to 29.4	37.2 (2.4) 33.7 to 42.5						8 (6, 11) 5 to 12	197.5 (94.5, 214.3) 28 to 237	5 (5, 6) 4 to 12	204 (175.3, 239.3) 139 to 503	120 (110, 120) 110 to 130	

*Note:* IVR, intravitreal injection of ranibizumab.

Abbreviations: AROP, aggressive retinopathy of prematurity; BW, body weight; CCH, cumulative clock hours; GA, gestational age; LP, laser photocoagulation; PMA, postmenstrual age; ROP, retinopathy of prematurity.

**Table 2:** Comparison of long-term follow-up physical examinations outcomes between 2 study groups.

Characteristic	Control group	LP + IVR group	*p* value
BW at birth, g, mean (SD)	1553.5 (392.7)	831.4 (140.9)	0.001^a∗^
GA at birth, week, mean (SD)	29.8 (1.4)	27.5 (1.7)	0.001^a∗^
Chronological age at physical examination, months, mean (SD)	29.8 (6.0)	30.0 (9.6)	0.797^a^
Ophthalmic outcomes			
BCVA, log MAR, mean (SD)	0.3 (0.1)	0.4 (0.1)	0.172^a^
SE, D, mean (SD)	+2.1 (0.6)	+1.9 (0.7)	0.086^a^
AL, mm, mean (SD)	21.6 (0.6)	21.9 (0.7)	0.140^a^
Somatic outcomes			
BW, kg, mean (SD)	15.1 (1.4)	14.8 (1.6)	0.608^a^
Body height, cm, mean (SD)	95.5 (5.4)	94.9 (4.3)	0.737^a^
Head circumference, cm, mean (SD)	48.2 (0.9)	47.9 (0.7)	0.279^a^
Developmental outcome (GDDS), M (P25, P75), range			
Gross motor	91 (85, 95) 77 to 100	89 (80, 93) 67 to 99	0.166^b^
Fine motor	92 (86, 95) 75 to 99	87 (84, 93) 69 to 99	0.154^b^
Adaptability	93 (88, 95) 76 to 99	88 (81, 94) 66 to 100	0.137^b^
Language	89 (84, 94) 67 to 99	87 (84, 92) 70 to 99	0.278^b^
Personal-social	91 (85, 96) 76 to 98	90 (84, 94) 77 to 98	0.391^b^

*Note:* IVR, intravitreal injection of ranibizumab.

Abbreviations: BW, body weight; CCH, cumulative clock hours; GA, gestational age; LP, laser photocoagulation; PMA, postmenstrual age; ROP, retinopathy of prematurity; SD, standard deviation.

^a^independent *t*-test.

^b^Wilcoxon rank-sum test.

^∗^ *p* < 0.05.

## Data Availability

All data examined in the current study can be provided by the corresponding author upon reasonable request.

## Supporting Information

Supporting Video 1: AL measurement in a 2-year-old using the NIDEK Optical Biometer.mp4. The video documented axial length measurement in a 2-year-old patient using the NIDEK Optical Biometer, illustrating patient positioning, device operation, and data acquisition.

## References

[1] Bremner A., Chan L. Y., Jones C., Shah S. P. (2023). Comparison of Weight-Gain-Based Prediction Models for Retinopathy of Prematurity in an Australian Population. *Journal of Ophthalmology*.

[2] Stahl A., Lepore D., Fielder A. (2019). Ranibizumab Versus Laser Therapy for the Treatment of Very Low Birthweight Infants With Retinopathy of Prematurity (RAINBOW): An Open-Label Randomised Controlled Trial. *Lancet*.

[3] Ghassemi F., Makateb A., Dastjani Farahani A., Mahmoudi A., Bazvand F. (2022). Evaluation of Neurodevelopmental Outcomes in Premature Twins (Multigestations) With Retinopathy of Prematurity Receiving Anti-VEGF: A Comparison Study. *Journal of Ophthalmology*.

[4] VanderVeen D. K., Melia M., Yang M. B., Hutchinson A. K., Wilson L. B., Lambert S. R. (2017). Anti-Vascular Endothelial Growth Factor Therapy for Primary Treatment of Type 1 Retinopathy of Prematurity. *Ophthalmology*.

[5] Palmer E. A., Flynn J. T., Hardy R. J. (2020). Incidence and Early Course of Retinopathy of Prematurity. *Ophthalmology*.

[6] Lu X., Zeng X., Chen M. (2022). Refractive and Biometrical Characteristics of Children With Retinopathy of Prematurity Who Received Laser Photocoagulation or Intravitreal Ranibizumab Injection. *Graefe’s Archive for Clinical and Experimental Ophthalmology*.

[7] Obata S., Matsumoto R., Iwasa M. (2023). Visual Field After Anti-Vascular Endothelial Growth Factor Therapy and Laser Treatment for Retinopathy of Prematurity. *Graefe’s Archive for Clinical and Experimental Ophthalmology*.

[8] Zeng X., Chen M., Zheng L. (2022). Study of the Biological Developmental Characteristics of the Eye in Children After Laser Surgery for the Treatment of Retinopathy of Prematurity. *Frontiers of Medicine*.

[9] Chen X., Zhou L., Zhang Q., Xu Y., Zhao P., Xia H. (2019). Serum Vascular Endothelial Growth Factor Levels Before and After Intravitreous Ranibizumab Injection for Retinopathy of Prematurity. *Journal of Ophthalmology*.

[10] Tian Y., Fan Z., Zeng X. (2024). Long-Term Follow-Up of the Cognitive Function in Children After Intravitreal Ranibizumab for Retinopathy of Prematurity. *Graefe’s Archive for Clinical and Experimental Ophthalmology*.

[11] Lyu J., Zhang Q., Chen C., Xu Y., Ji X., Zhao P. (2019). Ranibizumab Injection and Laser Photocoagulation to Treat Type 1 Retinopathy of Prematurity After 40 Weeks Post Menstrual Age: A Retrospective Case Series Study. *BMC Ophthalmology*.

[12] Sabancı Ş., Küçük M. F., Süren E., Erol M. K. (2024). Comparison of Intravitreal Bevacizumab Monotherapy and Combined Laser Photocoagulation and Intravitreal Bevacizumab Therapy in the Same Session in the Treatment of Aggressive Retinopathy of Prematurity. *International Ophthalmology*.

[13] Dabir S., Mohankumar A., Srivatsa D. V. (2023). Retinopathy of Prematurity in Preterm Infants Born Following Assisted Conception Versus Spontaneously Conceived Pregnancies—A 2-Year Retrospective Observational Study From an Urban Tertiary Eye Care Referral Center in South India. *Indian Journal of Ophthalmology*.

[14] Jang H. G., Choi S., Noh O. K., Hwang J. H., Lee J. H., Neonatal Network K. (2023). Comparison of Neonatal Outcomes Between Multiples and Singletons Among Very Low Birth Weight Infants: The Korean Neonatal Network Cohort Study. *Journal of Maternal-Fetal and Neonatal Medicine*.

[15] Friling R., Axer-Siegel R., Hersocovici Z., Weinberger D., Sirota L., Snir M. (2007). Retinopathy of Prematurity in Assisted Versus Natural Conception and Singleton Versus Multiple Births. *Ophthalmology*.

[16] Fierson W. M., American Academy of Pediatrics Section on Ophthalmology, American Academy of Ophthalmology, American Association for Pediatric Ophthalmology and Strabismus, American Association of Certified Orthoptists (2018). Screening Examination of Premature Infants for Retinopathy of Prematurity. *Pediatrics*.

[17] Huang Q., Zhang Q., Fei P. (2017). Ranibizumab Injection as Primary Treatment in Patients With Retinopathy of Prematurity: Anatomic Outcomes and Influencing Factors. *Ophthalmology*.

[18] Duelund N., Nisted I., Jørgensen M. E., Heegaard S., Jensen H. (2024). Vision Screening and Refraction of Greenlandic Schoolchildren. *Acta Ophthalmologica*.

[19] Stahl A., Nakanishi H., Lepore D. (2024). Intravitreal Aflibercept vs Laser Therapy for Retinopathy of Prematurity Two-Year Efficacy and Safety Outcomes in the Nonrandomized Controlled Trial FIREFLEYE next. *JAMA Network Open*.

[20] Yu X., Chen H., Savini G. (2018). Precision of a New Ocular Biometer in Children and Comparison With IOLMaster. *Scientific Reports*.

[21] Tian W., Zhao X., Xu H., Sun Y., Zhu M. (2024). Application of the Hammersmith Infant Neurological Examination in the Developmental Follow-Up of High-Risk Infants. *Developmental Medicine and Child Neurology*.

[22] Hartnett M. E., Toth C. A. (2019). Experimental Evidence Behind Clinical Trial Outcomes in Retinopathy of Prematurity. *Ophthalmic Surgery Lasers and Imaging Retina*.

[23] Kumar P., Sankar M. J., Deorari A. (2011). Risk Factors for Severe Retinopathy of Prematurity in Preterm Low Birth Weight Neonates. *Indian Journal of Pediatrics*.

[24] Chen J., Hao Q., Zhang J., Du Y., Chen H., Cheng X. (2023). The Efficacy and Ocular Safety Following Aflibercept, Conbercept, Ranibizumab, Bevacizumab, and Laser for Retinopathy of Prematurity: a Systematic Review and meta-analysis. *Italian Journal of Pediatrics*.

[25] Zhang G., Yang M., Zeng J. (2017). Comparison of Intravitreal Injection of Ranibizumab Versus Laser Therapy for Zone II Treatment-Requiring Retinopathy of Prematurity. *Retina*.

[26] Sanghi G., Dogra M. R., Katoch D., Gupta A. (2013). Aggressive Posterior Retinopathy of Prematurity: Risk Factors for Retinal Detachment Despite Confluent Laser Photocoagulation. *American Journal of Ophthalmology*.

[27] Dwivedi A., Dwivedi D., Lakhtakia S., Charudutt C. (2024). Anatomical Outcome of Laser Treatment Alone in Aggressive Retinopathy of Prematurity. *Oman Journal of Ophthalmology*.

[28] Katoch D., Dogra M. R., Aggarwal K. (2019). Posterior Zone I Retinopathy of Prematurity: Spectrum of Disease and Outcome After Laser Treatment. *Canadian Journal of Ophthalmology*.

[29] Lepore D., Quinn G. E., Molle F. (2018). Follow-Up to Age 4 Years of Treatment of Type 1 Retinopathy of Prematurity Intravitreal Bevacizumab Injection Versus Laser: Fluorescein Angiographic Findings. *Ophthalmology*.

[30] Chow S. C., Lam P. Y., Lam W. C., Fung N. S. K. (2022). The Role of Anti-Vascular Endothelial Growth Factor in Treatment of Retinopathy of Prematurity—A Current Review. *Eye*.

[31] Feng J., Qian J., Jiang Y. (2017). Efficacy of Primary Intravitreal Ranibizumab for Retinopathy of Prematurity in China. *Ophthalmology*.

[32] Gangwe A. B., Agrawal D., Gangrade A. K., Parchand S. M., Agrawal D., Azad R. V. (2021). Outcomes of Early Versus Deferred Laser After Intravitreal Ranibizumab in Aggressive Posterior Retinopathy of Prematurity. *Indian Journal of Ophthalmology*.

[33] Parchand S. M., Agrawal D., Gangwe A., Saraogi T., Agrawal D. (2021). Combined Intravitreal Ranibizumab and Zone I Sparing Laser Ablation in Infants With Posterior Zone I Retinopathy of Prematurity. *Indian Journal of Ophthalmology*.

[34] Hwang E. S., Kassem I. S., Allozi R. (2022). Association Between Myopia Progression and Quantity of Laser Treatment for Retinopathy of Prematurity. *PLoS One*.

[35] Chen Y. C., Chen S. N. (2020). Foveal Microvasculature, Refractive Errors, Optical Biometry and Their Correlations in School-Aged Children With Retinopathy of Prematurity After Intravitreal Antivascular Endothelial Growth Factors or Laser Photocoagulation. *British Journal of Ophthalmology*.

[36] Chou Y. B., Wang A. G., Yang H. Y., Chen K. J., Yang C. S. (2022). Refractive Status, Biometric Components, and Functional Outcomes of Patients With Threshold Retinopathy of Prematurity: Systemic Review and a 17-Year Longitudinal Study. *Graefe’s Archive for Clinical and Experimental Ophthalmology*.

[37] McLoone E. M., O’Keefe M., McLoone S. F., Lanigan B. M. (2006). Long-Term Refractive and Biometric Outcomes Following Diode Laser Therapy for Retinopathy of Prematurity. *Journal of American Association for Pediatric Ophthalmology and Strabismus*.

[38] Ruan L., Shan H. D., Liu X. Z., Huang X. (2015). Refractive Status of Chinese With Laser-Treated Retinopathy of Prematurity. *Optometry and Vision Science*.

[39] Young-Zvandasara T., Popiela M., Preston H., Seow E., Watts P. (2019). Is the Severity of Refractive Error Dependent on the Quantity and Extent of Retinal Laser Ablation for Retinopathy of Prematurity?. *Eye*.

[40] Alqurashi L., Alfaraidi A., Almahmoudi F., Danish E., Hadrawi M. (2023). Refractive Changes Among Diode Laser-Treated Retinopathy of Prematurity Patients: A Retrospective Study. *Middle East African Journal of Ophthalmology*.

[41] Hoppe C., Holt D. G., Arnold B. F., Thinda S., Padmanabhan S. P., Oatts J. T. (2022). Structural and Refractive Outcomes of Intravitreal Ranibizumab Followed by Laser Photocoagulation for Type 1 Retinopathy of Prematurity. *Journal of American Association for Pediatric Ophthalmology and Strabismus*.

[42] Murakami T., Okamoto F., Kinoshita T. (2023). Comparison of Long-Term Treatment Outcomes of Laser and Anti-VEGF Therapy in Retinopathy of Prematurity: A Multicentre Study From J-CREST Group. *Eye*.

[43] Christoforidis J. B., Briley K., Binzel K. (2017). Systemic Biodistribution and Intravitreal Pharmacokinetic Properties of Bevacizumab, Ranibizumab, and Aflibercept in a Nonhuman Primate Model. *Investigative Opthalmology & Visual Science*.

[44] Murakami T., Sugiura Y., Okamoto F. (2021). Comparison of 5-Year Safety and Efficacy of Laser Photocoagulation and Intravitreal Bevacizumab Injection in Retinopathy of Prematurity. *Graefes Archive for Clinical and Experimental Ophthalmology*.

